# A novel *G6PD* deleterious variant identified in three families with severe glucose-6-phosphate dehydrogenase deficiency

**DOI:** 10.1186/s12881-020-01090-2

**Published:** 2020-07-17

**Authors:** Yongqing Tong, Bei Liu, Hongyun Zheng, Anyu Bao, Zegang Wu, Jian Gu, Bi-Hua Tan, Mary McGrath, Shriya Kane, Chunhua Song, Yan Li

**Affiliations:** 1 Department of Clinical Laboratory, Renmin Hospital of Wuhan University, 99 Ziyang Road of Wuchang District, Wuhan, 430060 People’s Republic of China; 2 Department of Pathology, Affiliated Tianyou Hospital of Wuhan University of Science and Technology, Wuhan, 430064 People’s Republic of China; 3 Pennsylvinia State University Hershey Medical Center, 500 University Drive, Hershey, PA 17033 USA; 4 University: Georgetown University School of Medicine, Washington, DC 20007 USA

**Keywords:** D-G6PD, Variant, Neonatal jaundice, Infant

## Abstract

**Background:**

Glucose-6-phosphate dehydrogenase deficiency (D-G6PD) is an X-linked recessive disorder resulted from deleterious variants in the housekeeping gene Glucose-6-phosphate 1-dehydrogenase (*G6PD*), causing impaired response to oxidizing agents. Screening for new variations of the gene helps with early diagnosis of D-G6PD resulting in a reduction of disease related complications and ultimately increased life expectancy of the patients.

**Methods:**

One thousand five hundred sixty-five infants with pathological jaundice were screened for *G6PD* variants by Sanger sequencing all of the 13 exons, and the junctions of exons and introns of the *G6PD* gene.

**Results:**

We detected *G6PD* variants in 439 (28.1%) of the 1565 infants with pathological jaundice. In total, 9 types of *G6PD* variants were identified in our cohort; and a novel *G6PD* missense variant c.1118 T > C, p.Phe373Ser in exon 9 of the *G6PD* gene was detected in three families. Infants with this novel variant showed decreased activity of G6PD, severe anemia, and pathological jaundice, consistent with Class I *G6PD* deleterious variants. Analysis of the resulting protein’s structure revealed this novel variant affects G6PD protein stability, which could be responsible for the pathogenesis of D-G6PD in these patients.

**Conclusions:**

High rates of *G6PD* variants were detected in infants with pathological jaundice, and a novel Class I *G6PD* deleterious variants was identified in our cohort. Our data reveal that variant analysis is helpful for the diagnosis of D-G6PD in patients, and also for the expansion of the spectrum of known *G6PD* variants used for carrier detection and prenatal diagnosis.

## Background

Glucose-6-phosphate dehydrogenase (G6PD) is a cytosolic enzyme encoded by a housekeeping X-linked gene, the main function of this gene is to produce Nicotinamide Adenine Dinucleotide Phosphate (NADPH). NADPH is a key electron donor that protects against oxidizing agents and participate in reductive biosynthetic reactions [1, 2]. G6PD has extraordinary genetic diversity [1–3]. Many variants of *G6PD*, mostly generated from missense variants, have been reported to possess a various enzyme activity and associated clinical symptoms [2–6]. Glucose-6-phosphate dehydrogenase deficiency (D-G6PD), also named Favism, is the potential cause of clinical acute hemolysis, neonatal jaundice, or severe chronic non-spherocytic hemolytic anemia [2, 7–10].

**Fig. 1:**
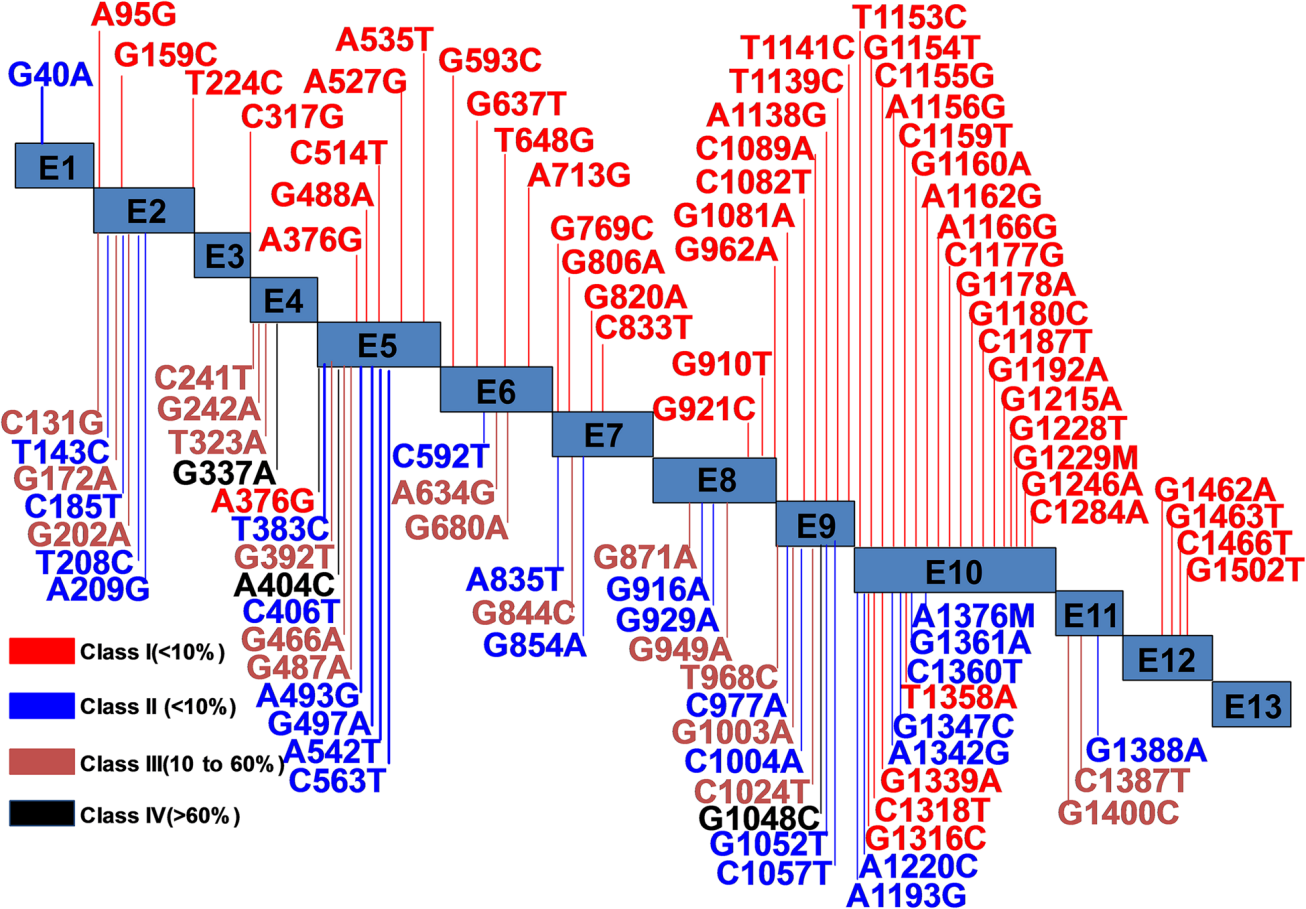
The common variants and classification in the *G6PD* gene. The *G6PD* gene variants are classified into Class I to Class IV based on the genotype and clinical manifestations (WHO classification). Red color indicates a Class I severe enzyme deficiency with chronic non-spherocytic hemolytic anemia (CNSHA). Blue color indicates a Class II severe enzyme deficiency with less than 10% of the normal activity. Orange color indicates a Class III mild to a moderate enzyme deficiency (10 to 60% of normal activity). Black color indicates a Class IV very mild or almost normal enzyme activity (> 60% normal activity and no clinical problem). Data from *G6PD* gene variant database (http://www.bioinf.org.uk)

More than 300 *G6PD* gene variants have been described in D-G6PD. These variants have been categorized into five classes, from Class I to Class V, by the World Health Organization (WHO) based on biochemical phenotype and clinical manifestations [11–13]. About 400 million people worldwide has been estimated to have D-G6PD [14]. This condition appears most frequently in certain parts of Africa, the Mediterranean, Asia, and the Middle East. Some genetic variants have reached a high incidence rate in people from certain parts of the world since they present with a selective advantage against malaria [15]. Mutations at different sites of the *G6PD* gene result in different effects on enzyme activity [16–19] (Fig. 1). The majority of variants of the *G6PD* gene result in red cell enzyme deficiency through decreasing enzyme stability [18, 19]. The polymorphic variants of the *G6PD* gene influence amino acid residues at multiple sites all over the enzyme and decrease the stability of the enzyme in the red cell, possibly by affecting protein folding [18–20]. These unfavorable variants of the *G6PD* gene mostly disturb residues at the dimer interface, or the residues responsible for association with a structural NADP molecule to stabilize the enzyme [21–25]. The de novo variants appear very rare, which causing the more severe condition of chronic non-spherocytic hemolytic anemia [20].

Pathological jaundice is an important condition and accounts for a large number of Neonatal Intensive Care Unit (NICU) admissions. Generally, jaundice in infants commonly presents in the first week of life. Pathological jaundice appears as early as the first day of life and can lead to adverse complications in the absence of timely intervention. A total serum bilirubin (TSB) level above the 95th percentile for an infant’s age (in hours) is defined as serum hyperbilirubinemia, which occurs in 8–9% of infants during the first week of life [26, 27]. The frequency of G6PD deficiency in infants with jaundice is well reported, however, the frequency of *G6PD* variants with G6PD deficiency in the infants with jaundice has not be studied to a great extent [28–30]. In this study, we screened for *G6PD* variants via DNA sequencing using blood samples from infants with pathological jaundice who were suspected to have D-G6P. We identified a new *G6PD* deleterious variant in three families with D-G6PD. Our data are indicative of the molecular mechanism underlying D-G6PD, and the importance of the molecular diagnosis and genetic screening for this disease.

## Methods

### Patient data and family consent

One thousand five hundred sixty-five infants born with pathologic jaundice at Renmin Hospital of Wuhan University between September 2015 to September 2018 were screened for *G6PD* gene variants. The identified novel variants were verified in 350 infants without jaundice as unrelated controls, and 80 blood donors serving as healthy controls were also screened. The red cell count (RBC), hemoglobin (Hb), hematocrit (HCT), total bilirubin (TBIL) and direct bilirubin (DBIL) were tested in all the newborns using routine clinical laboratory methods as previously reported [31–33]. Ethical Committee of Renmin Hospital of Wuhan University approved this study. Written consents were obtained from the families for reporting their clinical details.

### G6PD enzymatic activity detected

The improved G6PD Nitroblue tetrazolium (NBT) Quantification Ratio Kit (Micky, Guangzhou, China) was utilized to measure G6PD enzymatic activity on a microplate reader (BioTek ELx808, USA) according to the manufacturer’s instructions. Those with a G6PD /6PGD ratio of ≤1.0 were classified as G6PD deficient [31–33].

### Variant analysis with Sanger sequencing

Primer3 web version 4.1.0 (http://primer3.ut.ee/) was utilized to identify, select, and design primers specific to target regions for all the 13 exons and intron-exon boundaries (100 bp upstream and downstream of each exon) of *G6PD* (GenBank ID: NG_009015.2; https://www.ncbi.nlm.nih.gov/nuccore/NG_009015.2/) (Supplemental Table 1). The variants were analyzed in both *G6PD* splice variant 1 (NM_000402.4) (https://www.ncbi.nlm.nih.gov/nuccore/NM_000402), and its splice variant 2, which lacks exon 1 (NM_001042351.2) (https://www.ncbi.nlm.nih.gov/nuccore/NM_001042351.2), The *G6PD* gene has 3 promoters based on the Eukaryotic promoter database (https://epd.epfl.ch//index.php); the promoter2 sequence is also covered in the intro-exon1 region. The extracted genomic DNA was amplified by polymerase chain reaction (PCR) using *h*-Taq DNA polymerase (bioWORLD, Ohio, USA). Then, shrimp alkaline phosphatase (USB, Cleveland, OH, USA) and exonuclease I (USB, Cleveland, OH, USA) were sued for purification of PCR products. The BigDye Terminator version 3.1 Cycle Sequencing Kit (Applied Biosystems, Foster City, CA, USA) was used for the purified PCR products. The products were then sequenced with 3500xl Genetic Analyzer (Applied Biosystems, Foster City, CA, USA). Sequencing Analysis version 5.2 (Applied Biosystems, Foster City, CA, USA) and ChromasPro version 1.5 (Technelysium PtyLtd., Tewantin, QLD, Australia) software were utilized for collected sequencing data analysis.

### Bioinformatics analysis

Effect prediction of amino acid substitutions on protein function was performed by the bioinformatic tools Sorting Intolerant From Tolerant (SIFT) (http://sift.jcvi.org/), Polymorphism Phenotyping v2 (PolyPhen-2) (http://genetics.bwh.harvard.edu/pph2/index.shtml), and Mutation Taster (http://www.mutationtaster.org/). Splice-site prediction was also performed using Maximum Entropy Modeling of Short Sequence Motifs (Max-EntScan) (http://genes.mit.edu/burgelab/maxent/Xmaxentscan_scoreseq.html), Neural Network Splice site prediction (NNSPLICE) (http://www.fruitfly.org/seq_tools/splice.html), GeneSplicer (http://www.cbcb.umd.edu/software/genesplicer/) and Human Splicing Finder (http://www.umd.be/HSF/). The alignment tool MSA (Multiple Sequence Alignment, https://www.ebi.ac.uk/Tools/msa/clustalo/) was utlized for multiple sequence alignments of G6PD protein sequences from different species (mammals, fish, and reptiles). The conservation of the variant across species was assessed by Genomic Evolutionary Rate Profiling (GERP) scores (https://www.ebi.ac.uk/training/online/glossary/gerp-score), and Combined Annotation-dependent Depletion (CADD) scores (https://cadd.gs.washington.edu/score).

Protein stability analyses and structure predictions were carried out using the software Triple-Helical collage Building Script (The BuScr) 1.06 (http://structbio.biochem.dal.ca/jrainey/THeBuScr.html), SCWRL 4.0 (http://dunbrack.fccc.edu/Software.php) and UCSF Chimera 1.5 (http://www.cgl.ucsf.edu/chimera/).

## Results

### *G6PD* variants analyzed in infants with pathological jaundice

One thousand five hundred sixty-five infants with pathological jaundice had *G6PD* variant screening performed by PCR amplification of the exons of the *G6PD* genes following Sanger sequencing. As a control, more than 300 unrelated infants (without jaundice) were also screened. There was a 28.1% variant detection rate with 439 infants out of 1565 infants found to have 9 different *G6PD* genetic variants. Detailed characteristics of each *G6PD* gene variants in two spice form and the number of times detected are summarized in Table 1. We also calculated the proportion of each variants in the 439 positive infants (Table 1). The most detected *G6PD* variants are c.1388G > A, p.Arg463His (9.33%) and c.1376G > T, p.Arg459Leu (8.95%). These two variants were found in 33.26 and 31.89% of subjects respectively, and represent the majority of variants found in our cohort. The order of incidence for the other variants from most frequent to least frequent is: c.95A > G,p.His32Arg (3.19%) with a proportion of 11.39%, c.871G > A, p.Val291Met (2.04%) with a proportion of 7.29%, c.1024C > T, p.Leu342Phe (1.79%) with a proportion of 6.38%, c.466G > A, p.Glu156Lys (1.02%) with a proportion of 3.64%, and c.1192 G > A, p.Glu398Lys and c.1004 C > A, p.Ala335Asp with a very low incidence of 0.77%, and a proportion of 2.73%.

**Table 1:** The detection rate of *G6PD* variants in infants with pathologic jaundice

Nucleotide changed	Protein changed	No. of the detected variants	Variant proportion (*n* = 439)	Variant frequency (*n* = 1565)	ClinVar Variation ID	HGMD Accession Number
NM_001042351.2:c.95A > G NM_000402.4:c.185A > G	NP_001035810.1:p.His32Arg NP_000393.4:p.His62Arg	50	11.39%	3.19%	10,403	CM910158
NM_001042351.2:c.466G > A NM_000402.4:c.556G > A	NP_001035810.1:p.Glu156Lys NP_000393.4:p.Glu186Lys	16	3.64%	1.02%	10,364	CM880031
NM_001042351.2:c.871G > A NM_000402.4:c.961G > A	NP_001035810.1:p.Val291Met NP_000393.4:p.Val321Met	32	7.29%	2.04%	10,386	CM930275
NM_001042351.2:c.1004 C > A NM_000402.4:c.1094C > A	NP_001035810.1:p.Ala335Asp NP_000393.4:p.Ala365Asp	12	2.73%	0.77%	NA	CM950506
NM_001042351.2:c.1024C > T NM_000402.4:c.1114C > T	NP_001035810.1:p.Leu342Phe NP_000393.4:p.Leu372Phe	28	6.38%	1.79%	10,405	CM930276
NM_001042351.2:c.1118 T > C NM_000402.4: c.1208 T > C	NP_001035810.1:p.Phe373Ser NP_000393.4:p.Phe403Ser	3	0.68%	0.19%	NA	NA
NM_001042351.2:c.1192 G > A NM_000402.4:c.1282G > A	NP_001035810.1:p.Glu398 Lys NP_000393.4:p.Glu428Lys	12	2.73%	0.77%	NA	CM910162
NM_001042351.2:c.1376G > T NM_000402.4:c.1466G > T	NP_001035810.1:p.Arg459Leu NP_000393.4:p.Arg489Leu	140	31.89%	8.95%	100,058	CM910163
NM_001042351.2:c.1388G > A NM_000402.4:c.1478G > A	NP_001035810.1:p.Arg463His NP_000393.4:p.Arg493His	146	33.26%	9.33%	100,059	CM910164

Note: In the database of Exome Aggregation Consortium (ExAC), ClinVar and HGMD, some *G6PD* variants are only expressed in *G6PD* splice variant 1 (NM_000402.4) (https://www.ncbi.nlm.nih.gov/nuccore/NM_000402), some in splice variant 2, which lacks exon 1 (NM_001042351.2) (https://www.ncbi.nlm.nih.gov/nuccore/NM_001042351.2), and some in both splice variants. The *G6PD* mutant in the two splice variants are listed in the table. We were not able to amplify the first exon of the *G6PD* in the 1565 infants. This means we could only detected the *G6PD* splice variant 1 in the cohort. All the *G6PD* variants we detected in our cohort from the Wuhan area are in *G6PD* splice variant 2, which is also the reason why each *G6PD* mutant in splice variant 2 (NM_001042351.2) is listed first in the table

### Mapping and sequencing for the novel *G6PD gene* variant

**Fig. 2:**
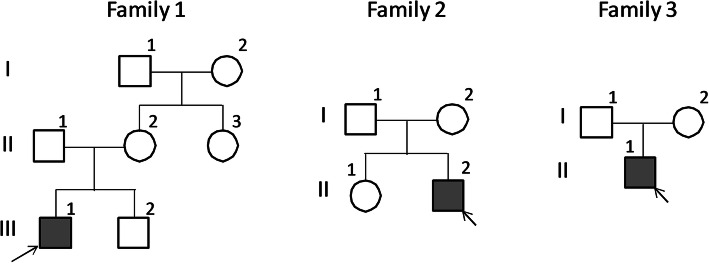
The pedigrees from three families. The filled symbols indicate affected individuals, the open symbols indicate unaffected individuals, the arrows indicate the index cases in each family, and the Roman numerals represent the generation numbers

**Fig. 3:**
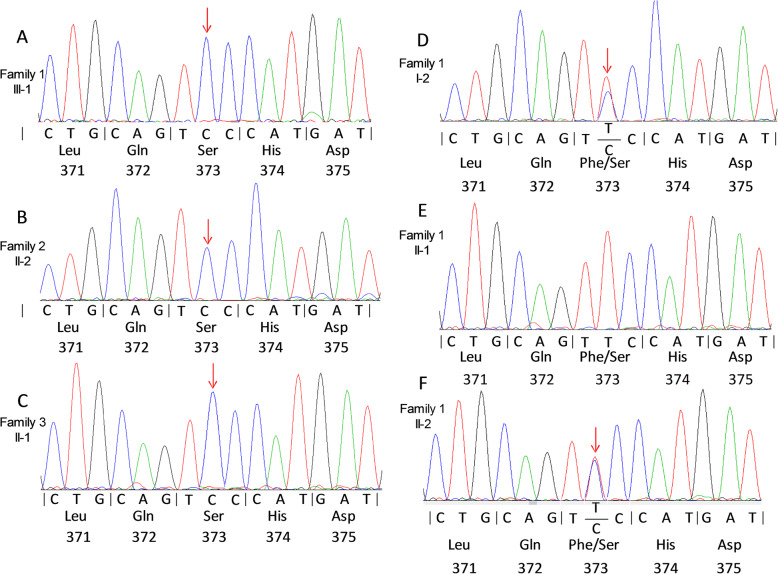
*G6PD* gene variant analysis in three families. **a**-**c** The sequence data are shown for the c.1118 T > C variant of *G6PD* gene exon 9 in the proband (II-1) of family1 (**a**), family 2 (II-2) (**b**) and family 3 (II-1) respectively. **d**-**f** the sequence data are presented for the c.1118 T > C variant in the mother or grandmother of the family1 (I-2) (**d**), (II-1) (**e**) and II-2 (**f**)

A novel *G6PD* variant, c.1118 T > C, p.Phe373Ser had the lowest incidence (0.19%) in our cohort with a proportion of 0.68% in our detected variants (Table 1). This variants appeared in 3 families, with a total of 14 family members affected (Family 1: 7affected; Family 2: 4 affected, Family 3: 3 affected) (Fig. 2). Analysis of all of the 13 exons of *G6PD* in the three probands when compared with the unrelated cohort revealed a hemizygous missense variant c.1118 T T > C in exon 9. This results in a putative amino acid change from phenylalanine (TTC) to serine (TCC) in codon 373 p.Phe373Ser (Fig. 3a~c). Exon 9 of the *G6PD* gene was detected in all the other members of these three families. It was noted that the missense variant c.1118 T G > C, p.Phe373Ser came from the mothers in the family and not the fathers (Fig. 3d~f) (data only showed the family 1). Moreover, c.1118 T T > C, p.Phe373Ser was not present in 350 unrelated infants or in 80 healthy controls. The CADD score showed the *G6PD* c.1118 T > C is a potentially pathogenic variant (CADD score 25.5). The GERP score showed that the *G6PD* c.1118 T is highly conserved (score is 5.82).

### Novel Class I *G6PD* gene variant identified in the infants from 3 families

To examine if the novel variants impact G6PD function we also examined RBC, Hb, HCT, TBIL, DBIL and the G6PD activity in all persons from the three families. Results are shown in Table 2. The G6PD activity in three probands was 3.5% (Family I III1), 4.6% (Family 2 II2) and 5.1% (family 3 (II1), which is below the 10% of normal activity (100%). The Hb values in the three probands was examined and severe anemia was indicated in the infants (Family 1 III1, 89 g/L; Family 2 II2, 54 g/L; Family 3 II1, 99 g/L), and the mothers of the infants in the three probands (Family 1 II2, 93 g/L; Family 2 I2, 109 g/L; Family 3 I2, 95 g/L). The TBIL and DBIL data indicated the presence of jaundice in the three probands (Family 1 III1, TBIL 158.1 unol/L, DBIL 19.5 unol/L; Family 2 II2, TBIL 57.2 unol/L, DBIL 10.9 unol/L; Family 3 II1, TBIL 154.27 unol/L, DBIL 17.5 unol/L).

**Table 2:** The clinical data of three families

Items	Family 1							Family 2				Family 3		
**patient**	I1	I2	II1	II2	II3	III1	III2	I1	I2	II1	II2	I1	I2	II1
**age**	61	59	39	36	27	2.5	0.5	36	37	8	1	32	29	0.5
**Clinical diagnosis**	N	CHM	N	CHM	N	D-G6PD	N	N	CHM	CHM	D-G6PD	N	CHM	D-G6PD
**G6PD activity (%)**	97.5	63.8	98.3	65.8	96.4	3.5	98.1	96.2	67.2	66.1	4.6	95.8	61.8	5.1
**RBC (10**^**12**^**/L)**	4.78	3.28	3.98	3.24	4.54	2.72	4.59	4.98	3.78	3.04	1.73	4.51	3.46	2.61
**Hb(g/L)**	155	95	129	93	148	89	134	133	109	97	54	146	95	99
**HCT (%)**	0.44	0.29	0.36	0.28	0.42	0.26	0.40	0.41	0.33	0.28	0.15	0.43	0.29	0.29
**TBIL (unol/L)**	17.39	10.1	14.7	11.62	8.70	158.1	13.1	5.9	21.9	17.0	57.2	18.65	6.76	154.27
**DBIL (unol/L)**	5.4	2.3	4.1	2.8	2.0	19.5	2.8	2.0	6.1	5.2	10.9	6.8	3.0	17.5

All the test results of the patients were from the clinical tests in the clinical laboratory. The clinical laboratory performs quality control every day to ensure the reliability and accuracy of the test results

*N* normal, *CHM* chronic hemolytic anemia, *RBC BRI* adult 3.8–5.1 10^12^/L, child 3.1–5.3 10^12^/L, *Hb BRI* adult 115–150 g/L, child 110–149 g/L, *HCT BRI* adult 0.35–0.45, child 0.40–0.50), *TBIL BRI* 2–22 unol/L, *DBIL BRI* 0–7.0 unol/L, *BRI* biological reference Interval, *RBC* red cell count, *Hb* hemoglobin, *HCT* hematocrit, *TBIL* total bilirubin, *DBIL* direct bilirubin

### Pathogenicity analysis of the novel *G6PD* mutant

With bioinformatics tools to predict the effect of amino acid substitutions on protein function, the missense variant c.1118 T T > C, p.Phe373Ser was classified as pathogenic (Table 3). MutPred prediction score for this mutant was 0.902, revealing that this variant was a deleterious variant and has the probability for a gain of disorder (statistically significant *p* = 0.007). MutPred2 prediction analysis showed that the amino acid substitution of this variant is pathogenic (score 0.885), affecting amino acids 137, 173 and 233, and also the eukaryotic linear motifs.

**Table 3:** Disease causing variant and phenotype in Chinese patients with D-G6PD and hemizygote missense variant in *G6PD*

Genome (h19)	DNA	Protein	known variant	SIFT		PROVEAN		Variant taster		PolyPhen-2	
			ExAC or 1000G	score	prediction	score	prediction	prediction	probability	Prediction	Confidence
Chr X: 153760951 T > C	c.1118 T > C	p.Phe373Ser	No reports	0.000	Damaging	−6.58	Deleterious	disease-causing	1.0	Probably damaging	0.996

We further analyzed if the c.1118 T T > C, p.Phe373Ser variant meet the pathogenic criteria of American College of Medical Genetics (ACMG) for the classification of variants [34]. Our data showed that the c.1118 T T > C, p.Phe373Ser variant meets the criteria of 2 pathogenic strong (PS): de novo variant confirmed in parents (PS2) and appeared in several members of 3 families with increased segregation (PS1), and 2 pathogenic moderate (PM): a novel missense change at an amino acid (PM5) localizing at the well-studied functional domain (PM1). In addition, this variant meets 2 pathogenic supporting (PP) criteria of ACMG: multiple lines of computational evidence supporting a deleterious effect of this variant on G6PD function (pp3), and the patients with this variant showing the D-G6PD (phenotypes) (pp4). Taken together, these analyses indicated that the missense variant c.1118 T T > C, p.Phe373Ser meets the pathogenic criteria of ACMG: 2 strong (PS1 and PS2) or 1 strong (PS1 and PS2) and 2 moderate (PM1 and PM5).

### Conservation and stability analysis of the novel *G6PD* mutant

**Fig. 4:**
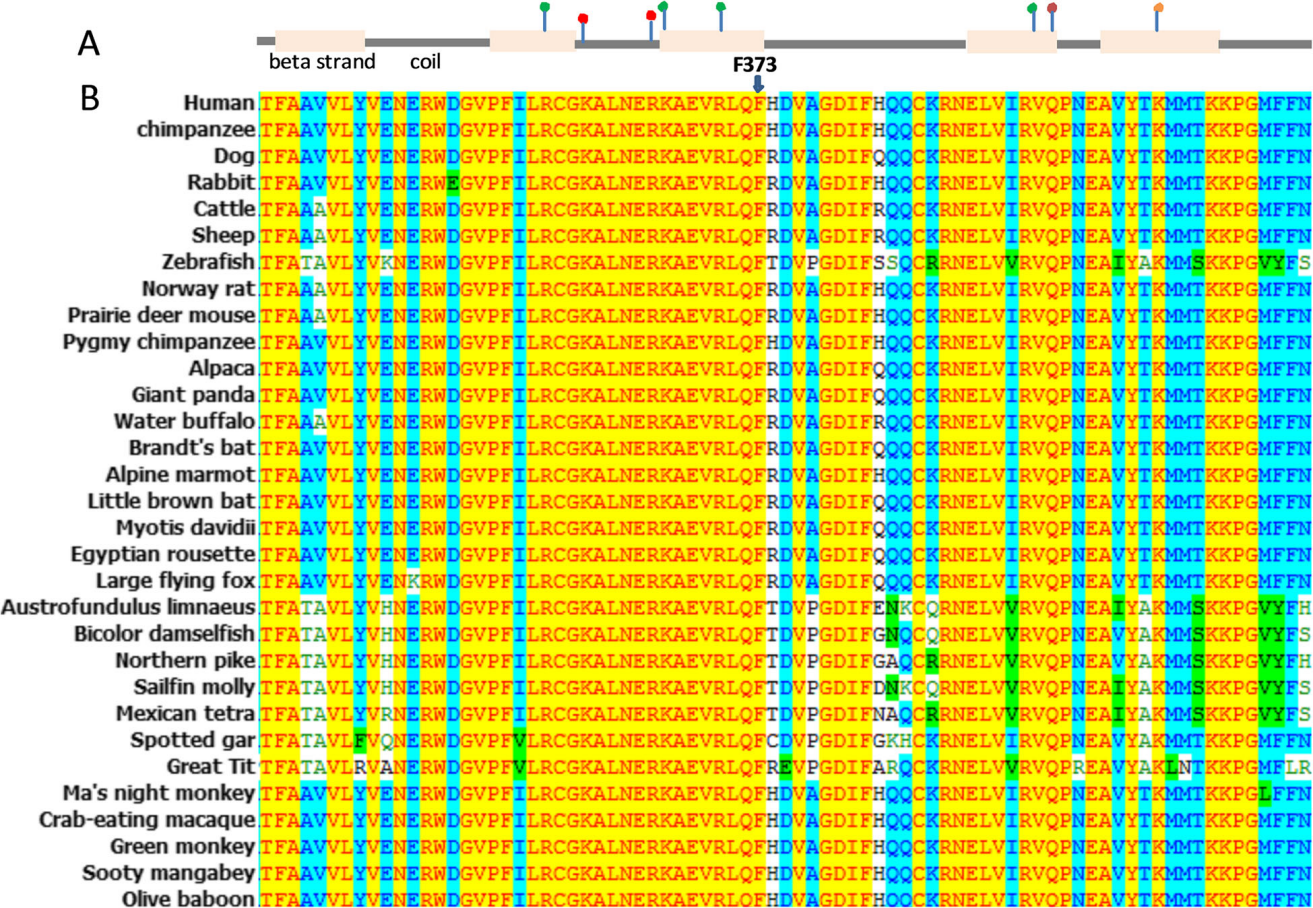
Evolutionary conservation of identified *G6PD* missense variant. **a** As shown, the second structure and p.Phe373 are located at the end of the beta strand, the green dot depicts the NADP binding site, the orange dot shows the N6-acetyllysine modified residue, and the red dot shows the substrate binding site. **b***G6PD* sequences from different species were aligned using the Clustal Omega in order to investigate the evolutionary conservation of the phenylalanine residue located in the codon. The multiple alignments revealed total conservation of this residue across all species, suggesting that this residue is crucial for the protein functionality

According to Alamut, both nucleotide c.1118 T and amino acid phenylalanine 373 are highly conserved. MultAlin Multiple sequence alignments of G6PD protein sequences from different species (Fig. 4) also showed high evolutionary conservation with respect to phenylalanine-373, which is substituted by serine in the affected members of the Chinese family.

**Fig. 5:**
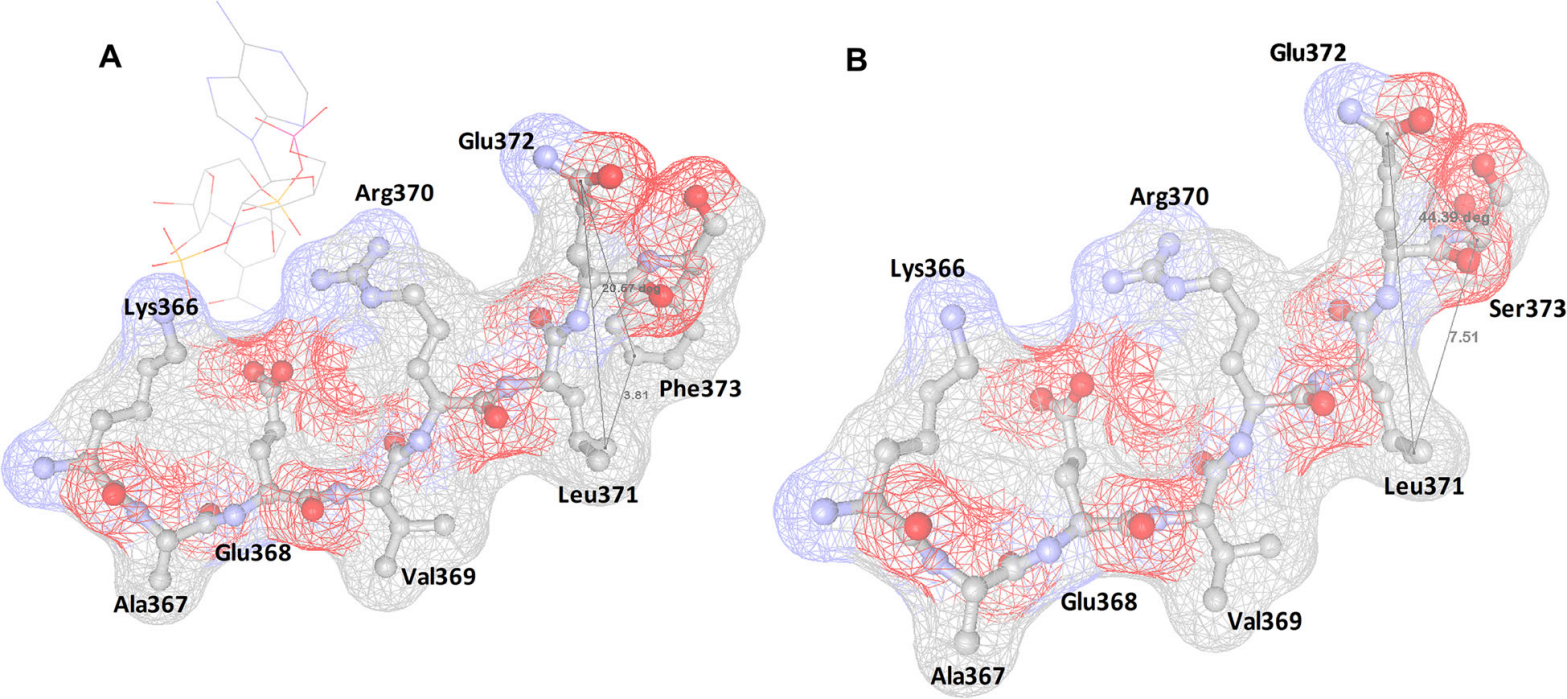
Modeling of the Phe373Ser mutant (F373S) in the protein G6PD (PDB code 2BH9). The figure was obtained using the software VMD. **a** The distance from Phe373 to Leu371 of 3.81 Å and the angle of 20.67 °C in the wildtype of Phe373 is shown. **b** The distance from Ser373 to Leu371 of 7.51 Å, and the angle of 44.39 °C in the variant of Phe373Ser is shown

The angle of the amino acid and distance of atoms around the Phe373 and Phe373Ser residue were studied using the software Swiss-PdbViewer (DeepView). When the phenylalanine residue was substituted by a serine residue, there was marked enlargement in the distance of the atoms from 3.81 Å (wild type) to 7.51 Å (variant type), and the angle of the three amino acid significantly expanded from 20.67 °C (wild type) to 44.39 °C (variant type) (Fig. 5).

The stability prediction for the variant and the properties of the structural environment along with its values for wildtype and mutant residues were also studied. SDM2 predicts this variant to have reduced stability (predicted pseudo ΔΔG:-1.54). I-Mutant2.0 predicts this variant to have decreased stability (ΔΔG: -2.32). AUTO–MUTE predicts this variant to have decreased stability (ΔΔG: − 1.97). ProMaya (Protein Mutant stAbilitY Analyzer) predicts this variant to have decreased stability (ΔΔG: − 1.97). MutPred2 predicts this variant to have altered stability(*P* = 0.02), and CUPSAT predicts this variant to be destabilizing (ΔΔG:-1.75). ProSMS predicts this variant to be destabilizing (probability 0.84).

## Discussion

Several factors play a role in infants developing pathological jaundice including the imbalance between production, conjugation, and elimination of bilirubin, environmental factors, and ethnicity [35]. G6PD deficiency is one of the common etiological factors for infant pathological jaundice and the *G6PD* variants are the major reason for the D-G6PD in China. We screened for *G6PD* gene variants in infants with pathologic jaundice in the Wuhan area and found that gene variants were detected in 28.1% of infants, which is comparable to 31.5% detection rate reported by Yazd in Neonatal Pathologic Hyperbilirubinemia [28]. Our data is the first report of the incidence of *G6PD* gene variants in infants with G6PD deficiency and pathological jaundice in the southeastern area of China.

Screening for the *G6PD* variants and prevention of the clinical manifestations of D-G6PD is essential for the public health. Many factors such as chemotherapeutic drugs, household and environmental agents trigger hemolytic anemia in patients with G6PD deficiency. The newborn screenings are usually performed 1 week after birth. This screening is helpful to prevent hemolysis prompted by treatment of infections, and other triggers. In addition, screening will allow for immediate treatment of severe anemia and hemolysis with resuscitation and erythrocyte transfusion. The *G6PD* variant test is the gold standard for the diagnosis of D-G6PD. Almost all newborns with a positive result by G6PD serology screening will have a *G6PD* variant detected. Therefore, prenatal diagnosis should be performed when parents have clinical symptoms of D-G6PD, suspected D-G6PD, or older children impacted by D-G6PD following birth. The screening results should be shared with the parents and necessary education on D-G6PD should be provided. All of these approaches might be excellent prophylactic measures in preventing hemolytic crises later in life for the infants. Our study identified a novel G6PD variant, which will increase the spectrum of *G6PD* variants for diagnosis of D6PD deficiency, carrier detection and prenatal diagnosis.

Several methods have been used for screening the *G6PD* variants, for example, *G6PD* variant detection array, multicolor melting curve analysis, etc. However, these methods are difficult to detect the novel *G6PD* variants. We used Sanger sequencing following the PCR-amplification of all *G6PD* exons and the exon-intron boundaries, which can detect both the known and unknown variants in the patients. Next-generation sequencing is also used for identifying novel *G6PD* variants, however it is not a time or cost effective method and is not suitable for large-scale screening. Our method is a simple, quick and economic screening method for the *G6PD* variants.

Different *G6PD* gene variants cause different levels of enzyme deficiencies and disease manifestations [36]. Our data showed that c.1388 G > A, c.1376 G > T and c.95 A > G were the three most common *G6PD* variants, accounting for 76.54% of the total disease alleles in this study cohort. The detection rate and proportion of each variant was similar to other regions in south China [31–33, 37, 38]. Two common variants (detection rate > 5%) and three low frequency variants (detection rate 1–5%) found in this study belong to one of the 5 class deletion variants, with 2 variants (C.95A > G and c. 1192G > A) in Class I, 2 variants ((c.1466G > T and c. 1388G > A) in Class II, and 2 variants (c.466G > A and c.871G > A) in Class III, all of which are reported to cause different degrees of enzyme deficiencies [33].

We also identified a novel *G6PD* variant, c.1118 T > C. Infants with this variant appear pathologically jaundice. Pathogenicity analysis showed this is a deleterious variant; and it is pathogenic. Conservation and stability analysis showed that this variant would reduce the stability of the G6PD protein. Infants with this variant had severe anemia, which showed the morphological characteristics of the nonspherocytic hemolytic anemia (data not shown). Therefore, the identified novel variants c.1118 T > C belongs to the Class I *G6PD*. This data provides evidence that this novel *G6PD* variant is a cause of nonspherocytic hemolytic anemia, and has significant clinical impact on the pathology of G6PD-D although the frequency of the variant is low in this cohort. In the future, the functional analysis of the novel variant will be performed, particularly evaluating the effect of the variant on G6PD protein stability and cellular activity. Combining the cellular effect of the novel variant with the clinical cohort study focusing on the novel variant will emphasize its role in pathogenesis of D-G6PD.

## Conclusions

High rates of *G6PD* variants are detected in infants with pathological jaundice, and a novel Class I *G6PD* variant has been identified in our cohort. Our data reveal that variant analysis is helpful for the diagnosis of D-G6PD and also in expanding the spectrum of *G6PD* variants evaluated for in carrier detection and prenatal diagnosis.

## Supplementary information

**Additional file 1 **: **Table S1.** The primers for amplification and sequencing the exons of *G6PD* gene.

## Data Availability

All data supporting the results reported in a published article can be found. The patient datasets for the current study are not publicly accessible in accordance with local health research ethics protocols; however, it may be available from the corresponding author. The accession numbers or direct web links and the full names of the data banks/repositories corresponding to all of the datasets obtained from web-based sources are given in the text and tables.

## Abbreviations

*G6PD*: Glucose-6-phosphate dehydrogenase
D-G6PD: Glucose-6-phosphate dehydrogenase deficiency
NADPH: Nicotinamide Adenine Dinucleotide Phosphate
RBC: Red cell count
Hb: Hemoglobin
HCT: Hematocrit
TBIL: Total bilirubin
DBIL: Direct bilirubin
NBT: Nitroblue tetrazolium
