# Supplementary material for: A novel G6PD deleterious variant identified in three families with severe glucose-6-phosphate dehydrogenase deficiency
Source: BMC Med Genet. 2020 Jul 17;21:150. doi: 10.1186/s12881-020-01090-2 (PMC7367331; doi:10.1186/s12881-020-01090-2)
Supplement: Supplementary file 1 — Additional file 1 : Table S1. The primers for amplification and sequencing the exons of G6PD gene. [file 12881_2020_1090_MOESM1_ESM.docx]

**Table 1 The primers for amplification and sequencing the exons of *G6PD*  gene**

| Exon(including intron-exon boundaries) | Primer from 5’ to 3’ | Amplicon length |
| --- | --- | --- |
| 1 | Forward : GGGAAACCGGACAGTAGGG  Reverse : GGTGCGGGGTATAAAGGGAT | 529bp |
| 2 | Forward : CAGCCGTTCACAAGGAGTGATT  Reverse : CCAGGTAGAGCCGGGATGAT | 421bp |
| 3-4 | Forward : TTGTGGCCCAGTAGTGATCCT  Reverse : TGACACCCAACTATGATTGGC | 593bp |
| 5 | Forward: : TGCTAAGATGGGGCTGAACC  Reverse : GGAAAGGCGGTGTTTCGTG | 554bp |
| 6 | Forward : GTGTTGAGCCAGAGGGTCATC  Reverse : AGGTGAGGCTCCTGAGTACCA | 384bp |
| 7 | Forward : ATGGTGCAGAACCTCATGGT  Reverse : CATAAAACCGTGGGGTGCTTG | 517bp |
| 8 | Forward : TGAGTCTTGCAGCTTGTCACT  Reverse : TGACTTCTCCGGGGTTGAGG | 315bp |
| 9 | Forward : GAAGCCCAAGTTGTCATGTCCC  Reverse : GACCAGTGCGTGAGTGTCTC | 561bp |
| 10 | Forward : ACTGAGACACTCACGCACTG  Reverse : AGGGAGCTTCACGTTCTGTG | 425bp |
| 11-12 | Forward : CCTGACCTACGGCAACAGAT  Reverse : TCTCTTCATCAGCTCGTCTGC | 538bp |
| 13 | Forward : GAGAAGCCCAAGCCCATCC  Reverse : TAGCTGGGCTCGGGTAGTAG | 388bp |
